# Identification and characterization of a salt stress-inducible zinc finger protein from *Festuca arundinacea*

**DOI:** 10.1186/1756-0500-5-66

**Published:** 2012-01-24

**Authors:** Ruth C Martin, Kira Glover-Cutter, James C Baldwin, James E Dombrowski

**Affiliations:** 1 USDA-ARS National Forage Seed Production Research Center, 3450 S.W. Campus Way, Corvallis, OR 97331; 2 Applied Technology Center, 1050 Forrer Blv., Kettering, OH 45429

## Abstract

**Background:**

Increased biotic and abiotic plant stresses due to climate change together with an expected global human population of over 9 billion by 2050 intensifies the demand for agricultural production on marginal lands. Soil salinity is one of the major abiotic stresses responsible for reduced crop productivity worldwide and the salinization of arable land has dramatically increased over the last few decades. Consequently, as land becomes less amenable for conventional agriculture, plants grown on marginal soils will be exposed to higher levels of soil salinity. Forage grasses are a critical component of feed used in livestock production worldwide, with many of these same species of grasses being utilized for lawns, erosion prevention, and recreation. Consequently, it is important to develop a better understanding of salt tolerance in forage and related grass species.

**Findings:**

A gene encoding a ZnF protein was identified during the analysis of a salt-stress suppression subtractive hybridization (SSH) expression library from the forage grass species *Festuca arundinacea*. The expression pattern of *FaZnF *was compared to that of the well characterized gene for delta 1-pyrroline-5-carboxylate synthetase (*P5CS*), a key enzyme in proline biosynthesis, which was also identified in the salt-stress SSH library. The *FaZnF *and *P5CS *genes were both up-regulated in response to salt and drought stresses suggesting a role in dehydration stress. *FaZnF *was also up-regulated in response to heat and wounding, suggesting that it might have a more general function in multiple abiotic stress responses. Additionally, potential downstream targets of FaZnF (a MAPK [Mitogen-Activated Protein Kinase], GST [Glutathione-S-Transferase] and lipoxygenase L2) were found to be up-regulated in calli overexpressing *FaZnF *when compared to control cell lines.

**Conclusions:**

This work provides evidence that FaZnF is an AN1/A20 zinc finger protein that is involved in the regulation of at least two pathways initiated by the salt stress response, thus furthering our understanding of the mechanisms of cellular action during a stress that is applicable to commercial crops worldwide.

## Introduction

With an expected population of over 9 billion by 2050 and added abiotic and biotic plant stresses due to climate change, there is an increased demand for agricultural production on marginal lands. Additionally, the salinization of arable land has dramatically increased over the last few decades [1,2]. Soil salinity is one of the major abiotic stresses responsible for reduced crop productivity worldwide [3]. As land becomes less amenable for conventional agriculture, plants grown on marginal soils will be exposed to higher levels of soil salinity. Forage grasses are a critical component of feed used in livestock production worldwide, with many of these same species of grasses being utilized for lawns, erosion prevention, and recreation. Consequently, it is important to develop a better understanding of salt tolerance in forage and related grass species.

Since plants are sessile, they have developed mechanisms that enable them to sense stressful environmental conditions and to elicit complex interactions between signaling molecules and pathways to adapt to various stresses. In the genomic era, new methods for looking at plant abiotic stress responses have evolved. The availability of the genome sequence and other resources such as microarrays, knockout mutants, and ease of transformation of the model dicot, *Arabidopsis*, greatly advanced our understanding of how plants respond to stress, including knowledge on the various components utilized in the signaling and response pathways. More recently, the availability of sequencing in other model species as well as some crop species facilitated the use of microarrays to analyze genes that are up- or down-regulated in response to specific stresses [4,5]. As sequencing became less cost prohibitive, high-resolution transcript profiling has been used to identify stress related genes and pathways in both monocot and dicot species [5]. In response to salt or dehydration stress, small molecules such as ABA and calcium are utilized by plants to induce various signalling cascades. These pathways use various proteins such as phospholipases, kinases, calmodulin, calcium-binding proteins and transcription factors to activate genes and pathways necessary for water-related stress tolerance (for reviews see: [2,6-11]) and are often targets for genetic modification to improve salt/drought tolerance in plants [12,13].

One particular class of proteins which are involved in plant response to abiotic stresses are zinc finger proteins. Zinc finger proteins were first characterized by a motif present in a protein (TFIIIA) that contained zinc and had the ability to bind DNA [14]. Members of the zinc finger transcription factors are characterized by the number and arrangement of cysteine (C) and histidine (H) within the zinc finger (C_2_H_2_, C_3_H, C_2_C_2_, C_3_HC_4_, C_2_HC_5_) in combination with other hydrophobic amino acids essential for stabilizing the zinc finger [14,15]. The first plant-specific zinc finger, identified in petunia, belonged to the C_2_H_2 _type zinc finger family and contained a plant specific "QALGGH" domain [16]. In Arabidopsis, data mining revealed 171 genes coding for C_2_H_2_-type zinc finger proteins, of which 77 contained the plant specific "QALGGH" domain [17]. Similarly, 189 C_2_H_2 _zinc finger proteins were data mined in rice, of which 26 were up-regulated in response to abiotic stress (drought, and/or salt and/or cold), while 21 genes were down-regulated during abiotic stress [18]. Several Arabidopsis C_2_H_2 _zinc-finger proteins function as transcription repressors during drought, cold and high salinity stress conditions [19]. Many C_2_H_2_-type zinc finger proteins have been shown to have a role in stress responses in plants [16,19-25], and many of the genes for these proteins, when overexpressed in Arabidopsis or tobacco, were shown to improve stress tolerance.

Another family of zinc finger proteins is characterized by the presence of A20 and AN1 zinc finger domains. The A20 domain was first identified in the A20 protein which was induced by a tumor-necrosis-factor in human endothelial cells [26]. The AN1 domain was initially identified as a putative zinc finger domain in an ubiquitin-like protein (AN1) from *Xenopus laevis *[27]. The first plant A20/AN1 zinc finger protein (OSISAP1) identified and characterized from rice, was shown to be induced by multiple stresses including cold, desiccation, salt, submergence, heavy metals, and injury, as well as ABA (Abscisic Acid). When this gene was overexpressed in tobacco, it conferred tolerance to multiple stresses (cold, dehydration and salt) during germination and early seedling growth stages [28]. Subsequently Rice Stress Associated Proteins (OsSAP 1-18) containing A20 and/or AN1 zinc fingers were identified by *in silico *analysis and compared to Arabidopsis A20/AN1 SAPs (AtSAP 1-10) [29]. There are 11 Rice SAPs (OsSAP1-11) that contain single A20 and AN1 zinc finger domains at the N and C termini, respectively. OsSAP12 contains two A20 zinc finger domains at the N-terminus and an AN1 zinc finger at the C terminus. There are several OsSAP proteins that only contain one (OsSAP13-15) or two (OsSAP16-17) AN1 zinc finger domains, while OsSAP18 only contains the A20 zinc finger domain. Arabidopsis has 10 SAPs (AtSAP1-10) which contain both the A20 and AN1 zinc finger domains, 3 SAPs (AtSAP11-13) with two AN1 zinc-finger domains and one SAP (AtSAP14) with a single AN1 zinc finger [29].

Expression analysis under different abiotic stressful conditions revealed that many of these genes were induced in response to salt and dehydration stress. Overexpression of several of these genes or homologs from other species has been shown to increase tolerance to one or more of various abiotic stresses (salt, drought, cold, and/or heat) [28,30-33], but in one case overexpression increased tolerance to cold, but increased sensitivity to salt and drought [34]. Overexpression of *AtSAP10 *confers tolerance to heavy metals (nickel, manganese, zinc) and high temperature stress [35]. This zinc finger family of proteins shows potential for increasing or stabilizing crop production on marginal soils and during increasing abiotic stress conditions due to climate change.

In this paper, we describe the characterization of a gene encoding an A20/AN1 zinc-finger protein that was identified during the analysis of a salt-stress suppression subtractive hybridization (SSH) expression library in *F. arundinacea*, a moderately salt tolerant glycophyte http://www.salinitymanagement.org/Salinity%20Management%20Guide/cp/cp_7_table-1.html[36]. The expression pattern of *FaZnF *was compared to the well characterized dehydration stress tolerance gene, delta 1-pyrroline-5-carboxylate synthetase (*P5CS*). Expression levels of both genes were analyzed in response to different levels of salinity and to seven other forms of abiotic stress. *F. arundinacea *calli, which were transformed to over-express *FaZnF*, were used to identify potential downstream targets of FaZnF. In this report we present evidence that *FaZnF *plays a role in dehydration stress responses and is also responsive to heat and wounding stress in tall fescue.

## Materials and methods

### Plant materials

*F. arundinacea *(tall fescue) seeds were planted in 4-inch square pots (volume approximately 750 mL) in SB40 Sunshine Growing Mix (Sun Gro Horticulture, Canada). Plants were fertilized weekly using Technigro 20-18-20 all-purpose fertilizer (Sun Gro Horticulture, Canada). Plants were grown in a Conviron E15 (Conviron, Winnipeg, Canada) growth chamber under an 8-hr photoperiod at 21°C day and 18°C night. Plants were grown for 6 weeks and pots containing 12-16 plants were then used for various stress treatments described below. At the designated times, the aerial portions of 6-7 plants were collected, immediately frozen in liquid nitrogen and stored at -80°C for future analysis.

### Stress treatments

#### Salt Stress

Plants were subjected to salt stress by treating the soil with 500 mL of 500 mM NaCl. Plants showed signs of mild wilting in the leaf blades after 1 hr. The aerial portions of 6-7 plants were collected 12 and 24 hr after salt treatment, immediately frozen in liquid nitrogen and stored at -80°C.

#### Salt concentration analysis

Plants were grown in soil for 6 weeks. Each pot containing 10-14 plants was treated with 500 mL of one of the following: 0 mM, 100 mM, 200 mM 300 mM, 400 mM or 500 mM NaCl salt solution. Plants treated with 0-300 mM NaCl did not show any visible signs of wilting over the 24 hr period, but plants treated with 400 and 500 mM NaCl showed signs of mild wilting but recovered within the 24-hr treatment period. The aerial portions of 6-7 plants were collected at 0, 12 and 24 hr, immediately frozen in liquid nitrogen and stored at -80°C.

#### Osmotic stress

Plants were subjected to osmotic stress by treating the soil with 500 mL of 12.87% polyethylene glycol 6000 solution (PEG). Plants showed mild wilting of leaf blades after 1 hr of treatment. The aerial portions of 6-7 plants were collected after 12 and 24 hr of stress treatment.

#### Wilt (Drought)

To induce drought stress, watering was stopped and pots were allowed to dry overnight. After 24 hr some very mild wilting was observed. Tissue was collected at 24 and 48 hr after watering was stopped.

#### UV Stress

Plants were laid on their side and irradiated for 5 min. Two hand-held 254-nm UV short-wave devices (Model UVG-11, Ultra-violet Products Inc, USA) were held 12-15 cm above the stems and leaves. Tissue limpness was observed after irradiation and samples from 6-8 plants were collected 1 and 8 hr post treatment.

#### Heat stress

Plants were subjected to 40°C in a Conviron E15 Growth Chamber to simulate heat stress. The plants were well watered and placed in a shallow pan of water to maintain adequate hydration during heat stress treatments. Tissue from 6-8 plants was collected after 2 and 8 hr of heat stress.

#### Wounding

Plants were mechanically wounded by closing a hemostat perpendicularly across the leaves and stems 3-5 times. Aerial plant tissue was collected 12 hr after wounding.

#### Cold stress

Plants were subjected to 4°C for 24 hr. Tissue was collected from plants after 24 hr in the cold.

#### Control tissue

Control tissue was collected from plants that were untreated and watered normally. All tissues were collected, immediately frozen in liquid nitrogen and stored at -80°C.

### Cloning of *FaZnF *gene and construction of *Agrobacterium *vector, pVec8.Ubi-ZnF

The 5' end of *FaZnF *was isolated previously with a partial clone from a tall fescue salt-stress Subtractive Suppression Hybridization (SSH) library. This library was constructed and analyzed as described in an earlier paper except tall fescue tissue was used instead of *L. temulentum *[37]. In the analysis of the tall fescue SSH library, we confirmed salt induced expression of selected genes by Northern analysis (Dombrowski and Baldwin, unpublished results). Genes were selected based on homology to genes described in other systems that were shown to be involved in salt stress. Northern analysis confirmed that *FaZnF *was up-regulated by salt stress.

RACE (rapid amplification of cDNA ends) was used to isolate the 3' end of the gene. Total RNA was isolated from leaf/crown tissues of *F. arundinacea *plants which had been subjected to a 20-hr salt stress treatment, using TRIzol reagent (Life Technologies, Gaithersburg, MD) following the manufacturer's instructions. RNA was reverse transcribed to cDNA using SuperScript III Reverse Transcriptase (Invitrogen, Carlsbad, CA) according to the manufacturer's protocol with the modified oligo(dT) primer (5'-GGCCACGCGTCGACTAGTACT_17_-3'). The resulting cDNA was used for PCR amplification in a 25 μl reaction containing 1× HotStar Taq Master Mix (Qiagen, Chatsworth, CA), 1 μl cDNA, and 0.4 μM of each primer (5'-GCCCCCAAAGGCCCAAGCAGGTG-3' and 5'-GGCCACGCGTCGACTAGTAC-3'). The PCR cycle was as follows: an initial denaturation at 94°C for 14 min; 7 cycles of 94°C for 1 min, 72°C for 2.25 min; 35 cycles of 94°C for 1 min, 67°C for 45 sec, 72°C for 1 min; with a final extension at 72°C for 10 min. The initial PCR was followed with a nested PCR reaction using primers (5'-CAACTGCCGGTGCGGGAACCTGTACCTC and 5'-GGCCACGCGTCGACTAGTAC-3') and the same cycling conditions as used for the primary PCR. A 510-base pair product was gel purified, ligated into the p-GEM-T Easy vector (Promega, Madison, WI), cloned and sequenced. A BLAST search confirmed high homology to the ZnF genes from other species.

Primers were designed using the 5' sequence information from the initial salt-stress clone and the sequence from 3' RACE to obtain the complete *FaZnF *gene. An initial PCR was performed using primers (5'-GGGCAGGTCAGAATTGCTCG-3' and 5'-CGATTACTAGTTACTATTACCGGTTGCG-3') at a concentration of 0.6 μM each with 1× HotStarTaq Master Mix (Qiagen) and 1 μl of cDNA. Reaction conditions were as follows: an initial denaturation at 94°C for 14 min followed by 35 cycles of 94°C for 1 min, 52°C for 1.5 min, 72°C for 1.5 min; with a final extension at 72°C for 10 min. This PCR reaction was followed by nested PCR using the primers (5'-ATGGATCCCGCCGGAGAG-3'; *Bam*H I site underlined) and (5'-ATGGTACCACAGATTACAGAGTGC-3'; *Kpn *I site underlined) at a concentration of 0.6 μM each with 1× HotStarTaq Master Mix (Qiagen) and 0.3 μl of the primary PCR product. Cycling parameters were as in the primary reaction with the exception that the annealing temperature was 59°C. An 848-bp product was gel purified, ligated into the p-GEM-T Easy vector (Promega), cloned and sequenced. A BLAST search confirmed high homology to other *ZnF *genes. The purified *FaZnF *clone was digested with *Kpn *I and *Bam*H I and ligated into the *Bam*H I/*Kpn *I digested pVec8.Ubi vector using T4 DNA ligase in 1× T4 ligase buffer (New England Biolabs, Ipswich, MA). The pVec8.Ubi vector was obtained from CSIRO (Commonwealth Scientific and Industrial Research Organisation; Australia) [38,39]. The final clone was sequenced to verify complete insertion into the vector.

RACE was also used to isolate the 5' and 3' regions of the *P5CS *partial clones identified by PCR-based SSH library analysis [37]. Total RNA from leaf/crown tissues of *L. temulentum *plants subjected to a 12-hr salt stress treatment was isolated using TRIzol reagent (Life Technologies, Gaithersburg, MD) following manufacturer's instructions. RNA was prepared for 5'RACE, and first-strand cDNA synthesis was performed with the GeneRacer™ Oligo dT Primer included with the SuperScript™ III RT module following the manufacturer's instructions (Invitrogen, Carlsbad, CA). For 5' RACE, the cDNA was amplified with a gene-specific primer (5'-TGCCTCTCGGAATAACAAGGTCAATCA-3') and the kit 5' primer. For 3' RACE, the cDNA was amplified with the kit 3' RACE primer and a gene specific primer (5'-TCGGCTGACATGGATATGGCAAAACG-3'). The PCR reaction conditions were as follows: 5 cycles of 98°C for 10 sec, 70°C for 5 sec (decreasing 2°C/cycle), 72°C for 1 min 30 sec; followed by 25 cycles of 98°C for 10 sec, 60°C for 5 sec, 72°C for 1 min 30 sec; followed by a final extension at 72°C for 10 min. PCR products were purified and sequenced. Based on these sequences, primers were designed to amplify the coding sequence with an extension homologous to the vector (underlined) at each end (5'-CGACTCTAGAGGATCCATGGGCAGGGGAGGCATCGGA-3' and 5'-CGGTACCCGGGGATCCGAATCCTCTACCTGCAATCAATG-3') to facilitate In-fusion PCR Cloning (Clonetech, Ca) for future experiments.

### Phylogenetic analysis of *FaZnF*

The FaZnF cDNA and protein sequences were subjected to BLAST searches at NCBI (National Center for Biotechnology Information; http://blast.ncbi.nlm.nih.gov/) against the nr (nonredundant) nucleotide and protein databases to provide annotation information and ortholog sequences from other species [40,41]. Closely related DNA and protein sequences, the top alignment from each genera, were used for phylogenetic analysis and tree construction at the "Phylogeny.fr" website [42]. Additionally, the Stress Associated Protein (SAP) sequences from rice and *Arabidopsis *and the most closely related *Brachypodium *sequences containing at least one of each of the A20/AN1 domains were used for a phylogenetic analysis.

### Probe construction

Primer3 software [43] was used to design primers for *P5CS *probe synthesis (5'-CATCAAGACCCTCTGTCTTG-3' and 5'-GTATATTCTGGGATAATGACAG-3') based on the *LtP5CS *sequence. RNA from salt stressed tall fescue tissue was used to produce cDNA, as described above, which was used for PCR to produce an ~1.2 kb DIG-labeled *P5CS *probe using the PCR DIG Probe Synthesis Kit (Roche-Applied Science, IN) following the manufacturer's instructions. The PCR reaction conditions were as follows: an initial denaturation at 94°C for 4 min; followed by 30 cycles of 94°C for 30 sec, 55°C for 30 sec, 72°C for 1.0 min; with a final extension at 72°C for 10 min. Probe was used at 2 μl/mL of hybridization solution (Note: *FaP5CS *and *LtP5CS *sequences have 96% homology in 1.1 kb of this region; data not shown).

Primer3 software [43] was used to design primers for *ZnF *probe synthesis (5'-CGAGGGCTTTCTCGTATCAGTA-3' and 5'-GAGTGCTAGCTAAATGCGAAGC-3') to produce an ~ 800 bp DIG-labeled *ZnF *probe using DIG labeled dUTP (Roche-Applied Science, IN) for PCR following the manufacturer's recommendations. ExTaq Polymerase (Takara, WI) was used with the following PCR reaction conditions: an initial denaturation at 95°C for 4 min; followed by 35 cycles of 94°C for 30 sec, 60°C for 30 sec, 72°C for 1.0 min; with a final extension at 72°C for 10 min. Probe was used at 2 μl/mL of hybridization solution.

### RNA gel blot analysis of genes

Genes coding for *P5CS *and *FaZnf *were subjected to further analysis by RNA blot analysis. Harvested tissue from *F. arundinacea *plants that had been subjected to various stresses, was ground to a powder in liquid nitrogen using a precooled mortar and pestle. Total RNA extractions from these ground tissues were performed using TRIzol reagent (Life Technologies, Gaithersburg, MD) following the manufacturer's instructions. Ten μg of total RNA isolated from selected/treated *F. arundinacea *plant tissue was electrophoretically separated on 1.2% denaturing formaldehyde agarose gels and blotted onto Hybond N+ nylon membranes [44]. Regions of selected genes were amplified by PCR using gene-specific primers and DIG labeled dUTP. The membranes were hybridized overnight with a DIG-labeled probe in a solution containing 15% SDS, 0.25 M NaPO_4 _pH 7.2, 1 mM EDTA, 0.5% blocking solution (Blocking solution: 10% Boehringer-Mannheim blocking reagent, 100 mM maleic acid pH 8.0, and 1 M NaCl). Filters were washed in a solution containing 1% SDS, 20 mM NaPO_4 _pH 7.2, and 1 mM EDTA at 60°C for three 30-min washes. The blots were then blocked and incubated with anti-digoxigenin-alkaline phosphatase antibodies and washed following the manufacturer's instructions (Roche; IN). The chemiluminescent substrate, CDP-*Star *(Roche; IN), was applied to the blots and light emission was detected on X-ray film.

### Transformation of suspension cells with *FaZnF*

#### Generation of tall fescue suspension cultured cell lines

Tall fescue (TF) suspension cultured cells were derived from calli induced when juvenile plants or seeds were cultured on Callus Induction Media D (CIM D: MS salts with 5 mg/L 2,4-D, 30 g sucrose, 8 g Phytagar and 110 mg/L Nitsch & Nitsch Vitamins). After 4 weeks of incubation, callus tissues generated from diced seed or explant material were visually selected and propagated by sub-culturing on the same medium every two weeks. After 2-3 months, the friable callus tissue that developed was transferred to 30 mL of liquid suspension induction medium (SIM: 4.3 g MS salts, 10 mg 2,4-D and 30 g sucrose) [45] in a 125-mL Erlenmeyer flask and placed on a rotary shaker (195 rpm) in the dark at RT. After 1-2 months, the established suspension cultured cell lines were transferred into maintenance medium (MM: MS basal medium containing/L; 5 mg 2,4-D, 30 g sucrose, 1 mg thiamine, 100 mg myo-inositol and 1 mM EDTA). All cell lines used in this study are over seven years old. TF suspension cell lines were maintained in 40 mL of medium in 125-mL Erlenmeyer flasks on orbital shakers (195 rpm) in the dark. Cells (10 mL) were sub-cultured every 7 days into 30 mL of fresh sterile medium.

#### Preparation of *Agrobacterium*

The *Agrobacterium *vector pVec8.Ubi-FaZnF was introduced into *Agrobacterium *AGL-1 using the freeze thaw method [46]. *Agrobacterium *AGL-1 containing pVec8.Ubi-FaZnF was cultured on YEP agar plates containing 30 μg/mL rifampicin, 80 μg/mL carbenicillin and 100 μg/mL spectinomycin and incubated at 28°C to obtain individual colonies. An individual colony was used for transformation studies following Gelvin's protocol [47] with a slight modification. Briefly AGL-1 containing pVec8.Ubi-FaZnF was grown overnight in YEP medium containing rifampicin at 30 μg/mL, carbenicillin at 80 μg/mL, and spectinomycin at 100 μg/mL. The next day, 0.5-1 mL of the *Agrobacterium *overnight culture was added to 50 mL of AB sucrose minimal medium containing rifampicin, carbenicillin and spectinomycin at the same concentration. After growing overnight at 28°C, the bacteria were centrifuged and resuspended in 50 mL induction medium containing 100 μM acetosyringone. The bacterial cultures were placed in two 50-mL conical Falcon tubes on a rocker shaker overnight at room temperature. Transformation was performed using *Agrobacterium *in the induction medium.

#### *Agrobacterium *mediated transformation of suspension cell cultures

Suspension cell cultures eight days after subculturing were used for transformation experiments. Cells were allowed to settle and then approximately 4 mL of cells was transferred from the flask to a sterile disposable 100 × 20 mm Petri dish (Fisher Cat. No. 0875711Z) for transformation. Excess media was removed from the plates, and 1 mL of *Agrobacterium *was added to the cells and allowed to sit for approximately 2 hr. Transformation efficiency was improved when 10 μl of 1 M NaOH and 50 μL of cysteine (Sigma C7880; St. Louis, MO.; Stock 20 mg/mL; final concentration 0.5 mg/mL) were added as two droplets to a clear area of each original plate. An additional 1 mL of *Agrobacterium *culture was first mixed with the NaOH and cysteine solutions and then mixed with the suspension cells. The plates were then wrapped with Nescofilm (Karlan Reseach Products; Cottonwood, AZ) and placed in the dark at RT for three days. Following co-cultivation, 3 mL of MM medium was added to the plate and pipetted over the cells to loosen the cells and bacteria that were adhering to the plate. The medium was removed from the plate and the cells were washed with 2 mL of MM medium containing 400 mg/L timentin (GlaxoSmithKline; Research Triangle Park, NC) and 40 mg/L L-cysteine. This medium was removed and replaced with 10 mL of the same medium. Cultures were grown at room temperature in the dark on an Innova platform shaker at 80 rpm. Three days later, hygromycin (Cat. #10687-010; Invitrogen; Carlsbad, CA) was added to a final concentration of 40 mg/L to select for transformants. The culture medium was removed each week and replaced with fresh media. After approximately 4-6 weeks, the transformed cells grew into larger clusters which were removed and placed on solid MM2 media containing 400 mg/L timentin, 40 mg/L hygromycin, 40 mg/L cysteine and 3 g/L gelrite (Research Products International; Mt. Prospect, IL). After approximately 2-4 weeks, clusters that grew were spread thinly on new plates and were allowed to grow for 2 weeks to maximize the possibility that the selected clusters were derived from a single transformation event. A single homogenous culture representing each original cluster was selected and grown up as an individual line. Five independent lines overexpressing FaZnF and two non-transformed control lines were used for the Reverse Transcription quantitative PCR experiments (RT qPCR).

### RNA extraction and cDNA synthesis

Two non-transformed control calli (obtained from the same original suspension cell culture used for transformation) and five FaZnF overexpressing calli were harvested for RNA extraction. Briefly, 0.5 g of calli was submerged in Trizol and homogenized for 30 seconds (Ultra-Turrax^® ^T25 at a setting of 4). Samples were then frozen at -80°C, quickly thawed at 37°C, and centrifuged for 5 min at ~12500 × g to remove cellular debris. Continuation of RNA extraction followed the manufacturer's protocol for Trizol (Invitrogen, Carlsbad, Ca). After RNA purification, samples were treated with Turbo DNase following the manufacturer's instructions (Ambion, Austin, TX), and cDNA was synthesized using 5 μg of RNA from each sample with the SUPERSCRIPT III RT kit (Invitrogen, Carlsbad, CA) primed with random hexamers.

### RT qPCR analysis

Based on contig sequences for each potential stress-associated target gene (MYC, lipoxygenase L2, Gst24, and eIF1) and sequences of reference genes GAPDH and UBC for *F. arundinacea *from the TIGR database, specific qPCR primers were designed (Table 1). Two reference genes were used in this study to reduce the possibility that the selected genes might themselves be changing with overexpression of *FAZnF *and to increase confidence in the fold changes of other genes relative to reference genes. Sequences of all contigs/genes used in this paper are reported in Supplementary Figure 1. Roche LightCycler Probe Design Software 2.0 was used to design primers with an average melting temperature of 62°C that, when used for PCR, would produce a product between 75 and 125 bp. Quantitative PCR was performed in 20 μl reactions in 96-well plates with BioRad iQSyber Supermix using a BioRad iQ5 Real-time PCR detection system (Bio-Rad Laboratories, Hercules, CA). Serial dilution standard curves from pooled cDNA samples were utilized to test the efficiency and specificity of each primer (Table 1). The efficiency calculation is based on the slope of the serially diluted points: E = -1 + 10^(-1/slope)^, and the specificity of each primer was analyzed by its single point dissociation curve. The close range of efficiencies between the targets and controls allowed for a ΔΔCT analysis using GAPDH or UBC as calibrators [48]. Calculation of relative mRNA followed the equation: 2^(-ΔCtSample - ΔCtCalibrator) ^as described by Livak and Schmittgen [49]. For each 20 μl reaction, 0.5 μl of sample cDNA was used. Analysis was performed from five independently transformed biological replicates of overexpressing calli and repeated on three independent qPCR plates. Reactions were performed with the following PCR cycle: Initial denaturation for 3 min at 95°C; 50 cycles of 15 sec at 95°C, 1 minute at 59°C; followed by a dissociation curve. Error was calculated as Standard Error of the Mean (SEM).

**Table 1:** Gene target information.

Name	Top Reference Alignment	Arabidopsis Homolog	Position	Forward primer Reverse primer 5'-3'	% efficiency
*eIF1*	ref|XP_478516.1| translational initiation factor eIF1 *Oryza sativa*	AT1G20010	97	GAAGAACGTCTCAAATTTCCTCG CAGTTGCTCAGAAACCATGAATC	99

*GST24*	ref|XP_463733.1| glutathione S-transferase GST 24 | *Oryza sativa*	AT1G10370	80	AGAAGATCTCACCAACAAGAGC TCGCCGTGGAGGAGAAC	95

*Lipoxy-genase L2*	ref|XP_469655.1| lipoxygenase L-2; *Oryza sativa*	AT1G72520	361	CACGAGCCTGCCATTGATTA TGTGGTTGTTCTTGACGATGA	98

*MAPK1*	ref|XP_470659.1| Putative MAP kinase 1 *Oryza sativa*	AT3G45640	148	CCACGGAGAATTTGATAAAGGAAATAC TCCATCAGATTATTCGCTCAAATCAAG	95

*FaZnF*	TIGR Transcript Assembly TA567_4606 Putative Zn finger *Festuca arundinacea*	n/a	632	TGTGCTACCTCACCGTCA TCAGGATGCCCAACAACTAGA	96

*GAPDH*	TIGR Transcript Assembly TA626_4606 Glycerol aldehyde dehydrogenase *Festuca arundinacea*	n/a	951	ATGGGTTATGTTGAGGAGGATT TTGACGAAGTTGTCGTTCAGAG	99

*UBC*	TIGR Transcript Assembly DT703874 Ubiquitin conjugating enzyme *Festuca arundinacea*	n/a	239	CGGCGGCTTCAACTACA CTCGCCAGCATAGAGTG	102

Quantitative PCR primers for each gene and the relative position of the amplicon (center) in the Tall Fescue genes/contigs are indicated. Amplicon length was designed to be between 75-125 bp. Gene target references are also noted along with the PCR efficiency of each primer set. Where applicable, the corresponding Arabidopsis gene reference number of genes found to be up-regulated in *Arabidopsis *plants overexpressing OsSAP11 [32] are included.

**Figure 1:**
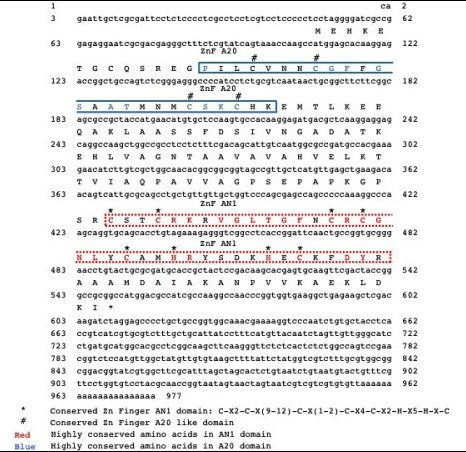
***Festuca arundinacea *zinc finger protein**. cDNA with translated protein shown above with functional regions of the zinc finger protein designated. Asterisks (*) indicate the conserved AN1 zinc finger domain (C-X2-C-X(9-12)-C-X(1-2)-C-X4-C-X2-H-X5-H-X-C); number symbols (#) indicate a conserved A20-like zinc finger domain. The solid red outlined box indicates the highly conserved amino acids in the AN1 domain and the blue dotted box represents the highly conserved amino acids in the A20 domain.

## Results and discussion

### Cloning and sequence analysis of the ZnF protein gene from *F. arundinacea*

Suppression subtractive hybridization (SSH) was used to identify differentially expressed genes related to salt stress in *F. arundinacea*. Analysis of the SSH library (SSH) revealed a partial clone coding for a ZnF protein. The preliminary screening of selected genes by RNA blot analysis confirmed that *FaZnF *was up-regulated by salt stress. BLAST search results indicated high homology to a transcription factor. Since transcription factors have the potential to regulate plant responses to multiple stresses and given the high homology of FaZnF to stress associated proteins across species, we focused our attention on this gene/protein as a possible transcription factor capable of directing the salt stress response in tall fescue.

**Figure 2:**
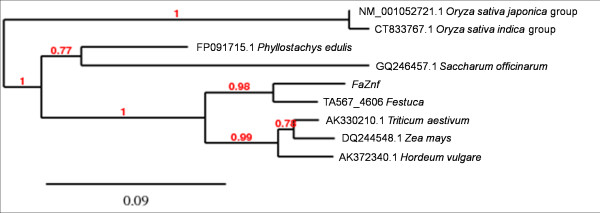
**Phylogenetic tree of *FaZnF *and related genes**. The top BLAST hits from each genera obtained when *FaZnF *was used for BLAST analysis at NCBI [49]. Also included is "TA567_4606_Festuca" which was the top BLAST hit when TIGR Plant Transcript Assembly http://plantta.jcvi.org/search.shtml was queried with *FaZnF *[50]. This transcript assembly contained ESTs from a heat stressed cDNA subtraction library from tall fescue [80] and was most closely related to *FaZnF*. The scale bar represents .09 nucleotide substitutions per site, or 9 nucleotide differences per 100 nucleotides.

**Figure 3:**
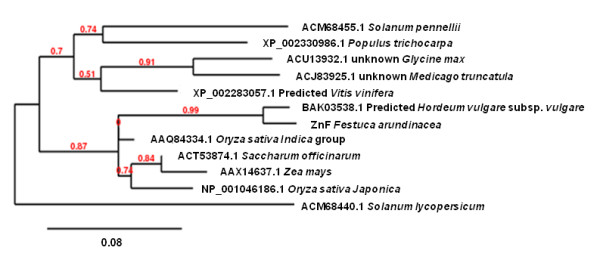
**Phylogenetic tree of FaZnF and related proteins**. The top BLAST hits from different genera (only the top hit from each genera is included) obtained when FaZnF was used for a protein BLAST analysis at NCBI [50]. The protein most closely related to FaZnF is a predicted protein from *Hordeum vulgare *which was identified in a subtraction cDNA library from seedling shoot and root treated with ABA. The scale bar represents .08 amino acid substitutions per site, or 8 amino acid differences per 100 amino acids.

**Figure 4:**
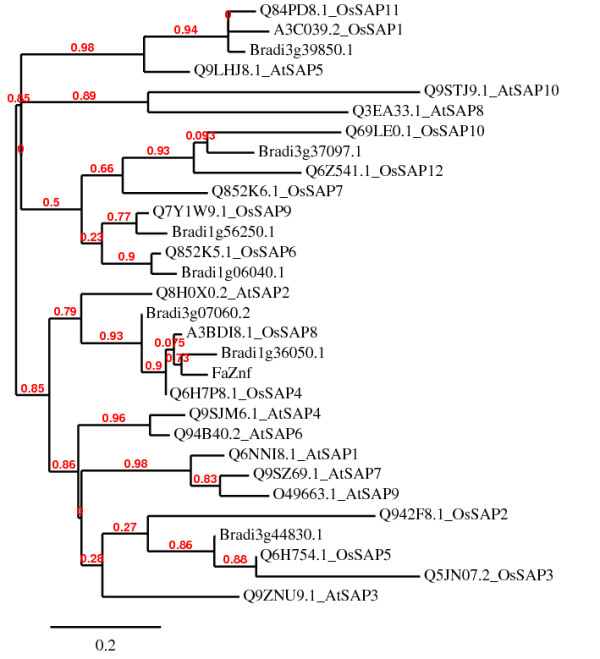
**Alignment of Stress Associated Proteins**. Alignment of Stress Associated Proteins (SAP) with both AN1/A20 domains from rice and *Arabidopsis *and most closely AN1/A20 domain-containing proteins from *Brachypodium*. FaZnF is most closely related to *Brachypodium *Bradi1g36050.1 and OsSAP8. The scale bar represent 0.2 amino acid substitutions per site, indicating 2 amino acid differences per 10 amino acids.

The whole gene sequence was obtained using 3' RACE (See Figure 1; Genbank Accession JN790818, to be released in April). Sequence analysis showed a full-length cDNA of 977 nucleotides, containing an open reading frame (ORF) of 501 nucleotides (ATG, 108-110; TAG, 609-611). To determine the function of this gene, this cDNA sequence was subjected to a BLAST search [50] against the nr nucleotide collection at the NCBI database and phylogenetic trees were constructed. The first gene listed on the BLAST results to *FaZnF *was from *Triticum aestivum *accession AK330210. The second gene on the list was a gene identified in osmotically stressed maize seedlings (Accession DQ244548) (See Phylogenetic Tree, Figure 2). Also included in the phylogenetic tree is "TA567_4606_*Festuca*" which was the top hit when *FaZnF *was subject to a BLAST search against the TIGR Plant Transcript Assemblies [51]. The identified ORF encoded a protein of 165 amino acids, with a potential molecular mass of 17.6 kDa and a pI of 8.6 (Figure 1). Using a BLAST protein similarity search, an additional phylogenetic tree was constructed [40]. The protein was most closely related to a predicted protein from *Hordeum vulgare *subsp *vulgare *(Accession number BAK03538.1) and the OsSAP8 from rice (Os06g0612800) (See Figure 3). Finally, to identify the most closely related stress associated zinc fingers containing at least one of each of the A20/AN1 domains from rice (OsSAPs) and *Arabidopsis *(AtSAPs) as well as the most closely related *Brachypodium *A20/AN1 genes (*Brachypodium distachyon *Acc ##; Bradi##), a phylogenetic tree was constructed. From this phylogenetic analysis, FaZnF was shown to be most closely related to *Brachypodium *Bradi1g36050.1, OsSAP 8 and 4, and AtSAP2 (See Figure 4) [42]. A gene closely related to *OsSAP8 *that was isolated from indica rice (*OsiSAP8*) has been characterized extensively and was also induced in response to multiple abiotic stresses including salt, drought, temperature, desiccation, submergence, wounding, heavy metals and ABA [52]. An OsiSAP8/GFP fusion protein was shown to be localized to the cytoplasm and the A20 and AN1 zinc finger domains were shown to interact using the yeast two-hybrid system suggesting that this zinc finger protein may function via protein-protein interactions [52].

### Response of *FaZnF *to salt stress

**Figure 5:**
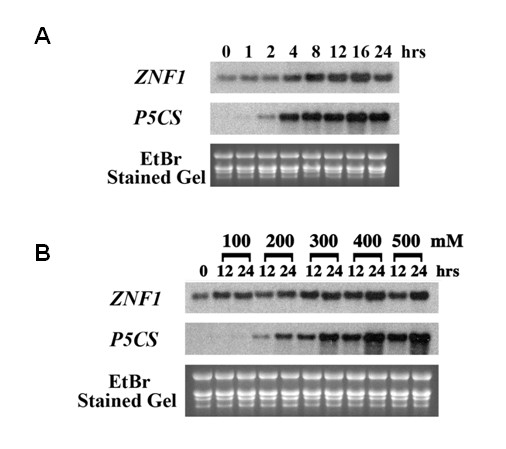
**Northern Blot analysis of *FaZnF *and *P5CS *expression**. (A) Northern Blot analysis of *FaZnF *and *P5CS *expression in response to salt stress for various time periods (0-24 hr). Total RNA was extracted from the aerial portions of plants that had been treated with 500 mM NaCl for the indicated number of hours. (B) Analysis of transcript levels of *FaZnF *and *P5CS *from plants treated with different levels of NaCl (0-500 mM) for 12 or 24 hr. Total RNA was extracted from the aerial portions of the plants that had been treated with different levels of NaCl (100-500 mM) for 12 or 24 hr, or with water (0 hr control). Ten μg of total RNA from each treatment was separated on a denaturing (formaldehyde) agarose gel. Equal loading was confirmed by visualization of the rRNA bands with ethidium bromide before (gel) and after transfer to the nylon membrane (blot). The RNA blots were hybridized with DIG-labeled DNA probes for *ZNF1 *and *P5CS*.

Since the *FaZnF *gene was initially isolated from salt stressed *F. arundinacea *plants, we wanted to investigate the kinetics of salt induced expression of this gene compared to the well characterized dehydration stress tolerance gene encoding a key enzyme in proline biosynthesis, delta 1-pyrroline-5-carboxylate synthetase (*P5CS*) [53]. Proline is an important osmoprotectant produced in plants in response to water related stresses [54-57] and recently was shown to be increased in tall fescue cultivars under drought stress conditions [58]. The *P5CS *gene has been shown previously to be involved in the biosynthesis of compatible solutes in response to salt stress in other plant species. Northern analysis of these two transcripts (*FaZnF *and *P5CS*) was performed in plants exposed to 500 mM NaCl over a 24 hr period. As shown in Figure 5A, *FaZnF *displayed a low level constitutive expression prior to salt stress (0 hr) and remained relatively constant until it started increasing at 4 hr post-stress induction and continued to increase through 16 hr and then decreased slightly from the 16-hr level to the 24-hr level. *P5CS *was not detectable prior to salt induction and started to increase slightly at 2 hr post-induction, gradually increasing to a maximum at 16 hr post-induction and slightly decreasing from that level at 24 hr.

To investigate the sensitivity of *FaZnF *and *P5CS *to different levels of salt stress, eight-week-old mature plants were treated with increasing concentrations of NaCl for 12 and 24 hr, and gene expression was assessed by Northern blot analysis. As shown in Figure 5B, *FaZnF *was expressed at a low level in the absence of salt (0), increased slightly at 12 and 24 hr with both 100 and 200 mM salt, but was increased greatly at 300, 400 and 500 mM NaCl, indicating that FaZnF has a role in the salt stress response in tall fescue. *P5CS *started to show induction at 12 and 24 hr with 200 mM salt and increased steadily as the salt concentration increased to 500 mM.

### The effect of different abiotic stresses on gene expression

**Figure 6:**
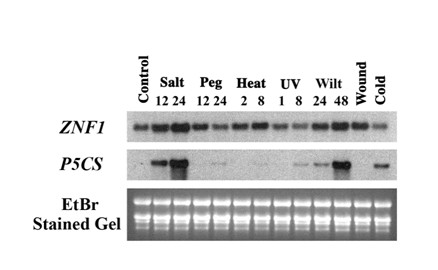
**Gene expression analysis of *FaZnF *and *P5CS *after exposure to different abiotic stresses**. Total RNA was extracted from the aerial portions of control plants (untreated and watered normally), from plants that had been subjected to different stresses for the indicated time periods, and from plants at specific time points after exposure to indicated stress (Salt: 500 mM NaCl; PEG: 12.87% PEG 6000; Heat: 40°C; UV: time after 5 min exposure to short-wave ultraviolet light; Wilting (Drought): no water for 24 or 48 hr; Wounding: 12 hr after mechanically wounded 3-5 times perpendicularly across the leaves; Cold: 4°C for 24 hr. Ten μg of total RNA from each treatment was separated on a denaturing (formaldehyde) agarose gel. Equal loading was confirmed by visualization of the rRNA bands with ethidium bromide before (gel) and after transfer to the nylon membrane (blot). The RNA blots were hybridized with DIG-labeled DNA probes for *ZNF1 *and *P5CS *genes.

The expression patterns of *P5CS *and *FaZnF *genes were examined in response to various abiotic stress conditions. In Figure 6, as shown in the previous figures, both genes were activated by treatment with 500 mM NaCl for 12 and 24 hr. PEG treatments resulted in a slight elevation of the *FaZnF *gene at 12 hr, which was back to control levels at 24 hr. *FaZnF *gene was induced by heat (moderate increase at 8 hr), wilting (slight increase at 24 hr, greatly increased at 48 hr) and wounding similar to *OsiSAP8*, but was not induced by cold or UV whereas *OsiSAP8 *was cold induced [52]. *OsSAP4 *was also shown to be induced by salt and dehydration [29]. Similar to most *SAP *genes that have been studied in plants, *FaZnF *was induced by multiple abiotic stresses. *P5CS *showed a slight induction 8 hr post UV treatment, during wilting (greatly increased at 48 hr), and was slightly increased after cold stress.

### Identification of possible targets of FaZnF

By identifying conserved domains within FaZnF, potential targets can be predicted using published target lists of orthologous proteins. To identify possible targets of FaZnf, SMART analysis was utilized to predict conserved protein domains http://smart.embl-heidelberg.de/. From this analysis, two specific zinc finger domains were identified including motif A20 (SMART accession SM00259) and zinc finger motif AN1 (SMART accession SM00154) with e-values of 1.53e-09 and 5.15e-16, respectively (Figure 1).

Some SAP proteins in Arabidopsis and rice have been localized to the nucleus [32,35] suggesting possible roles as transcription factors. Given the considerable amount of literature on A20/AN1 stress associated proteins (SAPs) in the model organism *Arabidopsis*, including microarrays documenting transcriptional changes in transgenic *Arabidopsis *overexpressing rice *OsSAP11 *or a protein that interacts with *OsSAP11 *(*OsRLCK253*) [32], we were able to compare a known Arabidposis A20/AN1 target list to the list of genes identified in the tall fescue salt SSH library. Of the 447 contig sequences from our salt SSH library, 38 sequences closely matched the Arabidopsis A20/AN1 data set through top reference alignment similarities. Confirmation of homology was then performed by tBLASTx analysis to Arabidopsis (SDSC workbench). Four representative contig sequences, each representative of a specific arm of the salt stress response, were identified and analyzed to determine if overexpression of *FaZnF *influences transcriptional activation; *MAPK, GST24, lipoxygenase L-2 *and *eIF1*.

### MAPK gene expression is elevated in calli overexpressing FaZnF

**Figure 7:**
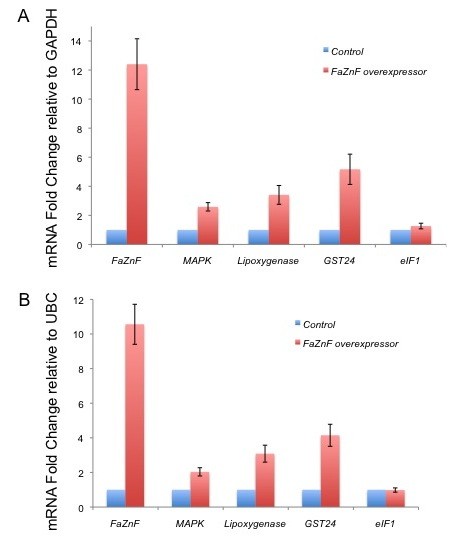
**Overexpression of FaZnF increases transcription of selected salt stress genes**. **A) **RT qPCR analysis of *Festuca arundinacea *calli using ΔΔCt quantification with GAPDH as the housekeeping gene. Values are normalized to control sample values (non-overexpressing calli) in order to represent relative expression changes. Error bars represent the SEM (Standard Error of the Mean). **B) **RT qPCR analysis of *Festuca arundinacea *calli using Ct quantification with UBC as the housekeeping gene. Values are normalized to control sample values (non-overexpressing calli) in order to represent relative expression changes. Error bars represent the SEM. Note that both housekeeping gene normalizations maintained the same trends in fold induction.

Signal transduction through MAPK activation during salt stress is essential for activation of the high osmolarity glycerol pathway (HOG) [59] which in turn enables survival during high osmotic stress [60]. Though MAPK is post-transcriptionally activated through a cascade of phosphorylation events, transcriptional induction also occurs during salt stress in *Arabidopsis *[61]. Consistent with these observations, our salt subtraction library also indicates that MAPK transcription is induced with salt stress in *F. arundinacea*. However, the general mechanism of transcriptional activation of the MAPK gene during salt stress is not clear. To address whether FaZnF can influence the transcription of *MAPK*, RNA was isolated from control calli and calli overexpressing FaZnF and changes in transcription were quantified by RT qPCR. Collectively, these overexpressing calli showed an increase in FaZnF expression by greater than 12-fold compared to housekeeping genes GAPDH or UBC (Figure 7). Interestingly, expression of *MAPK *was also increased almost 3-fold in overexpressing FaZnF calli, thus suggesting the possibility that the A20/AN1 domain containing FaZnF enzyme might regulate expression of *MAPK*, directly or indirectly.

### FaZnF influences transcription of oxidative stress pathway genes

Abiotic stressors such as salt stress increase the production of Reactive Oxygen Species (ROS), thus activating the oxidative stress pathway. Enzymes such as glutathione S-transferases are transcriptionally up-regulated to scavenge the elevated ROS to protect the organism [62]. Our salt subtraction library identified one major GST, *GsT24 *as being up-regulated with salt stress. Considering that overexpression of the highly homologous A20/AN1 SAP1 enzyme influenced transcription levels of many GSTs, experiments were performed to investigate whether FaZnF also influenced transcription of *GsT24*. Again, to determine transcriptional induction changes in mRNA, the expression of *GsT24 *was monitored in *F. arundinacea *control calli and FaZnF overexpressing calli by RT qPCR. Similar to *MAPK *expression, levels of *GsT24 *expression increased more than 5-fold in calli overexpressing FaZnF compared to control calli (Figure 7), strongly suggesting that this A20/AN1 protein influences the transcription of *GsT24*.

While combating oxidative stress, plants will utilize ROS as signaling molecules to either maximize the detoxification response or to accelerate cell death [62,63]. One way to intensify the production of ROS and potentially the oxidative stress response is through the up-regulation of lipoxygenases [64,65] which catalyze polyunsaturated fatty acid dioxygenation or pyridine nucleotide oxidation [66]. Many members of the lipoxygenase family are transcriptionally up-regulated with salt stress [67]. Similarly, lipoxygenase is up-regulated with salt stress in our salt SSH library, and overexpression of *FaZnF *results in a 3-fold increase in lipoxygenase L-2 transcript levels compared to housekeeping genes as determined by RT qPCR (Figure 7).

To ensure the most efficient use of cellular resources during salt or oxidative stress, translation is restricted [68,69]. Translation efficiency can be reduced by post-translational modification of the translation apparatus or through transcriptional regulation of key translation factors. However, similar to the heat shock stress response, the salt stress response can also be accompanied by salt stress recovery, where the cellular machinery anticipates increases in gene expression by increasing the transcription of translation factors [70]. Interestingly, overexpression of translation initiation factor eIF1A has been reported to increase salt tolerance in multiple organisms [71], and eIF1 was identified in our salt subtraction library as being up-regulated with salt stress. However, transcriptional analysis to determine whether overexpression of the A20/AN1 FaZnF results in increased *eIF1 *transcription did not show enhanced induction compared to housekeeping genes *GAPDH *or *UBC *(Figure 7). This result suggests that additional signals during salt stress might be required for activation of this translation initiation gene.

## Conclusions

In summary, one of the biggest challenges to agricultural yield worldwide is increased soil salinity [3,72]. Plants responding to increased salinity have at least two survival response strategies: activation of the MAPK pathway to deal with subsequent osmotic stress, and activation of the oxidative stress pathway to deal with large increases in reactive oxygen species (ROS). For many plants, including forage crops such as *F. arundinacea*, increased soil salinity induces osmotic and oxidative stress responses [73]. However, the cellular mechanisms driving these stress responses in grasses have not been fully delineated. Here we describe a highly conserved, from humans to plants, Stress Associated Protein (SAP) that contains the unique combination of dual zinc finger motifs, A20/AN1, in the forage grass *F. arundinacea*. This protein could act both as a transcription factor in the nucleus and as a mediator of stability and function in the mitochondria and cytoplasm, similar to the mammalian stress factors p53 and XBP1 [74-77]. This work provides evidence that FaZnF is involved in the regulation of at least two pathways initiated by the salt stress response. It is not yet known if the FaZnF protein acts through ubiquitin-related mechanisms like AtSAP5 [30] and animal ZNF216 proteins [78], or perhaps through interactions with protein kinases like OsSAP11/1 [32] and ZNF216 [79], or through interactions with other SAP/nonSAP proteins or transcription factors, or as a transcription factor itself.

Future studies are necessary to determine the cellular localization and mechanism of function for FaZnF, though it is quite possible that FaZnF has many functions as an abiotic stress factor, as do many stress related proteins. It will also be of additional interest to determine if overexpression of FaZnF will lead to greater abiotic stress tolerance in grasses.

## Abbreviations

ABA: abscisic acid; BLAST: basic local alignment search tool; CIM: callus induction medium; 2,4-D: 2,4-Dichlorophenoxyacetic acid; DNase: deoxyribonuclease; EDTA: ethylenediaminetetraacetic acid: FAO: Food and Agriculture Organization; FaZnF: *Festuca arundinacea *zinc finger protein; GST: glutathione-S-transferase; MAPK: mitogen-activated protein kinase; NaCl: sodium chloride; NaPO_4_: combination of NaH_2_PO_4 _and Na_2_HPO_4_; P5CS: 1-pyrroline-5-carboxylate synthetase; PCR: polymerase chain reaction; PEG: polyethylene glycol; RACE: random amplification of cDNA ends; ROS: reactive oxygen species; RT qPCR: reverse transcription quantitative PCR; SAP: stress associated protein; SDS: Sodium dodecyl sulfate; SSH: suppression subtraction hybridization; UV: ultraviolet.

## Competing interests

The authors declare that they have no competing interests.

## Authors' contributions

JED, JCB, and RCM conceived of the study and participated in its design. RCM was responsible for northern analysis and callus transformation. KGC designed and performed the RT qPCR analysis. RCM, KGC and JEB helped to draft the manuscript. All authors read and approved the final manuscript.

## Supplementary Material

Additional file 1 **Sequence of all genes/contigs used in this study are listed**. Protein sequence of the open reading frame of *FaZnF *is also included. Note that primers used for quantitative RT-PCR are highlighted in yellow. Contigs refer to the contig number of clones from the Tall Fescue salt SSH library that were sequenced. Click here for file
