# Supplementary material for: Identification and characterization of a salt stress-inducible zinc finger protein from Festuca arundinacea
Source: BMC Res Notes. 2012 Jan 24;5:66. doi: 10.1186/1756-0500-5-66 (PMC3305619; doi:10.1186/1756-0500-5-66)
Supplement: Additional file 1 — Sequence of all genes/contigs used in this study are listed. Protein sequence of the open reading frame of FaZnF is also included. Note that primers used for quantitative RT-PCR are highlighted in yellow. Contigs refer to the contig number of clones from the Tall Fescue salt SSH library that were sequenced. [file 1756-0500-5-66-S1.DOCX]

**Supplementary File 1**

Sequence of all genes used in this study. Protein sequence of the open reading frame of FaZnF is also included. Note that primers used for quantitative RT-PCR are highlighted in yellow. Contigs refer to the contig number of clones from the Tall Fescue salt SSH library that were sequenced.

>FaZnF cDNA sequence (To be released in Genbank in February: Acc #JN790818)

CAGAATTGCTCGCGATTCCTCTCCCCTCGCCTCCTCGTCCTCCCCCTCCTAGGGGATCGCCGGAGAGGAATCGCGACGAGGGCTTTCTCGTATCAGTAAACCAAGCCATGGAGCACAAGGAGACCGGCTGCCAGTCTCGGGAGGGCCCCATCCTCTGCGTCAATAACTGCGGCTTCTTCGGCAGCGCCGCTACCATGAACATGTGCTCCAAGTGCCACAAGGAGATGACGCTCAAGGAGGAGCAGGCCAAGCTGGCCGCCTCCTCTTTCGACAGCATTGTCAATGGCGCCGATGCCACGAAAGAACATCTTGTCGCTGGCAACACGGCGGCGGTAGCCGTTGCTCATGTTGAGCTGAAGACAACAGTCATTGCGCAGCCTGCTGTTGTTGCTGGTCCCAGCGAGCCAGCCCCCAAAGGCCCAAGCAGGTGCAGCACCTGTAGAAAGAGGGTCGGCCTCACCGGATTCAACTGCCGGTGCGGGAACCTGTACTGCGCGATGCACCGCTACTCCGACAAGCACGAGTGCAAGTTCGACTACCGGGCCGCGGCCATGGACGCCATCGCCAAGGCCAACCCGGTGGTGAAGGCTGAGAAGCTCGACAAGATCTAGGAGCCCCTGCTGCCGGTGGCAAACGAAAAGGTCCCAATCTGTGCTACCTCACCGTCATCGTGCGTCTTTGCTGCATTATCCTTTCATGTTACAATCTAGTTGTTGGGCATCCTGATGCATGGCACGCCTCGGCAAGCTTCAAGGGTTCTCTCACTCTCTGGCCAGTCCGAACGGTCTCCATGTTGGCTATGTTGTGTAAGCTTTTATTCTATGGTCGTCTTTGCGTGGCGGCGGACGGTATCGTGGCTTCGCATTTAGCTAGCACTCTGTAATCTGTAATGTACTGTTTCGTTCCTGGTGTCCTACGCAACCGGTAATAGTAACTAGTAATCGTCGTCGTGTGTTAAAAAAAAAAAAAAAAAAAAA

>FaZnF Protein (translation of cDNA)

MEHKETGCQSREGPILCVNNCGFFGSAATMNMCSKCHKEMTLKEEQAKLAASSFDSIVNGADATKEHLVAGNTAAVAVAHVELKTTVIAQPAVVAGPSEPAPKGPSRCSTCRKRVGLTGFNCRCGNLYCAMHRYSDKHECKFDYRAAAMDAIAKANPVVKAEKLDKI*

TF eIF1 Contiq Sequence 130:

>S6_SFA_Contig_130

ACTGTTGTCCAGGACTCAGAGCTTGGTCAGGTCATTCAACTTCAGGGTGACCAGAGGAAGAACGTCTCAAATTTCCTCGTCCAGGCCGGCATTGTGAAAAAGGAGCACATCAAGATTCATGGTTTCTGAGCAACTGCCAGCTCCATATCGAAGCTTGCATGCGAAGCAACTATATATTTGCAGTGGAGTATATTTGGTCGTGTGCTAGCAATAGCAGGCGTTACGCTTGCACGCTCTTAGTATACACAACAAAACCTAGCCCAGCCATCTCTCTGCGTGTATGCTTATCACCGTTGTGTCCAAACTTGT

TF Gst24 Contig Sequence 163:

>S6_SFA_Contig_163

GGCTCTGCGGCTCAAGGGAGTCCCCTTCGAGCTCGTCGTAGAAGATCTCACCAACAAGAGCGACCTCCTACTCACGCACAACCCCATCTACAAGACGGTCCCCGTTCTCCTCCACGGCGACCGGCCAGCCGTCTGCGAGTCGCTCATCATCGTGGAGT

TF Lipoxygenase L2 Contig Sequence 48:

>S6_SFA_Contig_48

GACAGGTAGCCATACTACCACAACCACTGTGGTTTTCCTCTCTTACAAGCACACACGAAGATGTTTGGCGGCGATATCATCAGCAACCTGACGGGAGGGCTCAAGAATGTCCACCTCAAGGGCTCTGTTGCCCTGATGCGCAAGAATGCGCTCGACTTCAACGATTTCGGCGCCGCTGCCATGGACAGCGTCACCGAGTTCCTCGGCCGTGGCGTCACCTGCCAGCTCATCAGCTCCACCGTCGTCGATTCCAACAACGGCAACCGCGGGAAGGTGGGCACCGAGGCGAGCCTGGAGCAGTGGATCACGAGCCTGCCATTGATTACGGTGGGCGAGTCCAAGTTCAAAGTCACGTTCGACTGGGACGTGGAGAAGATGGGGGTGCCCGGCGCGATCATCGTCAAGAACAACCACACCTCCGAGTTCTTCCTAAAGACCATCACCCTCGACGATGTCCCCGGCCGTGGCACCGTCCTCTTCGTCGCCAACTCATGGGTCTATCCCAAGGGCAACTACCGCTACAACCGCGTCTTCTTCGCCAACGATACGT

TF MAPK1 Contig Sequence 79:

>S6_SFA_Contig_79

ACGAGCAAGCCCAAAATCACAAATTTTTAGGTCACAGTTTGCATTCAAAAGAAGATTGCTAGGCTTCAAGTCTCGGTGGAGAACATTTGCTGAATGTATATACTTCAAGCCACGGAGAATTTGATAAAGGAAATACTGGCAATGTTCCTCCGATAAAGCTTGATTTGAGCGAATAATCTGATGGAGATCAGTGTCCATCAATTCCATATGCTATATAGACATCATTGAATGAACTCCTCTGTGCAGGAGGTATAATATCCCTTATTGCAACAATATTCTCGTGGTCCATGTGGCGAAGCAGCTTGATCTCCCGCAGCGTCCGCTTGGCGTCGATCTTGTTGTCGAAGGCGTTGGCGATCTTCTTGATGGCCACCTGCTCCCCCGTGTCGGAGTTGAGCGCGGAGCAGACGATGCCGTATGCGCCCTTGCCGATGGGGAGGATGGGGGGCTTGT

TF GAPDH sequence from TIGR:

TA626_4606

>TA626_4606

GGCACGAGGGACACTTCTCCCCACGCCGTCCAACATCTCCGTCTCGAGCGTCGTCACAGCTCCACTCGCGCCATGGGCAAGATTAAGATCGGAATCAACGGGTTCGGAAGGATCGGAAGGCTCGTCGCCAGGGTCGCCCTCCAGAGCGACGATGTCGAGCTCGTCGCAGTCAACGACCCCTTCATCACCACCGAGTACATGACCTACATGTTCAAGTACGACTCCGTGCACGGCCACTGGAAGCACAGCGACATCAAGCTCAAGAACGACAAGACTCTCCTCTTCGGCGAGAAGGCAGTTGCCGTCTTCGGCGTCAGGAACCCTGAGGAGATCCCATGGGCTGAGGCTGGTGCCGACTACGTCGTGGAGTCCACCGGTGTCTTCACTGACAAGGACAAGGCTGCTGCTCACTTGAAGGGTGGTGCCAAGAAGGTGGTCATCTCTGCTCCAAGCAAAGACGCCCCTATGTTCGTTGTTGGTGTCAATGAGGACAAGTACACTTCAGACGTTAACATTGTCTCAAATGCTAGCTGCACCACTAACTGTCTCGCTCCCCTAGCTAAGATCATTAATGACAACTTTGGTATTATTGAGGGTCTGATGACCACTGTTCATTCCATCACTGCCACCCAGAAGACTGTTGACGGACCCTCAAGCAAGGACTGGAGAGGTGGGAGGGCGGCAAGCTTCAACATCATCCCCAGCAGCACCGGCGCTGCCAAGGCTGTTGGCAAGGTTCTTCCTGAGTTGAATGGCAAGCTCACCGGTATGTCATTCCGGGTTCCCACAGTTGATGTGTCAGTTGTTGATCTCACCGTCAGAATTGAGAAGGCTGCATCATATGAGGACATCAAGAAGGCCATCAAGGCTGCATCTGAGGGAAACCTCAAGGGAATTATGGGTTATGTTGAGGAGGATTTGGTCTCCACTGACTTCATTGGTGATAGCAGGTCGAGCATCTTCGACGCCAAGGCTGGAATTGCTCTGAACGACAACTTCGTCAAGCTTGTCTCGTGGTACGACAACGAGTGGGGTTACAGCAACCGTGTCATCGACCTGATCCGCCACATGGCCAAGACACAGTAGAGTGTTCCGCGTTTCTTCTGGGCGAGGCCTGATCCGCCACATGGCCAAGACACAAGTAGTGTTCCGCTTCTCTTCTGGG

TF UBC sequence from TIGR:

DT703874

>DT703874

GGCACGAGGCACCTCACCGATACGCCAACCCGCACTCCCCACGTATCCTATTCCCCCTCTGCCTGCCTGCACCTTTCCCCTTCGGCGACCGCCCGCCCCATGACGAGCTCCTCCTCCCCTTCCCGGAAGGTGCTGAGCAAGATCGCCTGCAATCGGCTGCAGAAGGAGCTCGCCGAGTGGCAGGCCAGCCCTCCCGGCGGCTTCAACTACAAGGTCTCCGACAACCTCCAGAGGTGGGTCATCGAGGTGTCCGGCGCGGAGGGAACACTCTATGCTGGCGAGAAGTACCAGCTGCAGGTGGACTTCCCGGAGCATTATCCCATGGAGGCTCCGCAGGTTATCTTCATGAATCCGGCACCGATGCATCCGCATATTTACAGCAACGGGCACATCTGTCTAGATATATTGTATGACTCATGGTCGCCAGCAATGACGGTCAGTTCTGTATGCATCAGCATCTTGTCTATGTTGTCAAGTTCACCTGTGAAGGAACGCCCGGCTGATAATGACCGCTATGTGAGGAACT

Original FaZnF contig 355

>S6_SFA_Contig_355

CAGAATTGCTCGCGATTCCTCTCCCCTCGCCTCCTCGTCCTCCCCCTCCTAGGGGATCGC

CGGAGAGGAATCGCGACGAGGGCTTTCTCGTATCAGTAAACCAAGCCATGGAGCACAAGG

AGACCGGCTGCCAGTCTCGGGAGGGCCCCATCCTCTGCGTCAATAACTGCGGCTTCTTCG

GCAGCGCCGCTACCATGAACATGTGCTCCAAGTGCCACAAGGAGATGACGCTCAAGGAGG

AGCAGGCCAAGCTGGCCGCCTCCTCTTTCGACAGCATTGTCAATGGCGCCGATGCCACGA

AAGAACATCTTGTCGCTGGCAACACGGCGGCGGTAGCCGTTGCTCATGTTGAGCTGAAGA

CAACAGTCATTGCGCAGCCTGCTGTTGTTGCTGGTCCCAGCGAGCCAGCCCCCAAAGGCC

CAAGCAGGTGCAGCACCTGTAGAAAGAGGGTCGGCCTCACCGGATTCAACTGCCGGTGCG

GGAACCTGT
